# Real-world practice patterns for dry eye diagnosis: a multicenter observational study in Taiwan

**DOI:** 10.1007/s10384-025-01175-6

**Published:** 2025-03-08

**Authors:** Shu-Wen Chang, Shiuh-Liang Hsu, Chih-Chien Hsu

**Affiliations:** 1 Far Eastern Memorial Hospital, New Taipei City, Taiwan; 2 National Taiwan University Hospital, Taipei, Taiwan; 3 Kaohsiung Medical University Hospital, Kaohsiung, Taiwan; 4 Kaohsiung Medical University, Kaohsiung, Taiwan; 5 Kaohsiung Medical University Gangshan Hospital, Kaohsiung, Taiwan; 6 Taipei Veterans General Hospital, Taipei City, Taiwan; 7 College of Medicine, National Yang Ming Chiao Tung University, Taipei, Taiwan

**Keywords:** Dry eye disease, Cross-sectional study, Taiwan, Asia Dry Eye Society (ADES) guidelines

## Abstract

**Purpose:**

To investigate the concordance between Asia Dry Eye Society (ADES) diagnostic criteria and real-world hospital-based practice, and to analyze the clinical characteristics of patients with dry eye disease (DED), in Taiwan.

**Study Design:**

Noninterventional, cross-sectional, retrospective clinical study.

**Methods:**

Data were analyzed for adults with newly diagnosed DED from three tertiary hospitals. The primary endpoint was the proportion of patients diagnosed with DED who fulfilled ADES criteria for DED. Other outcomes were DED classification and severity, tear breakup time (TBUT), Schirmer’s test, corneal fluorescein staining (CFS) severity, 12-item Ocular Surface Disease Index (OSDI) questionnaire scores, and presence of meibomian gland dysfunction (MGD).

**Results:**

A total of 213 patients, mean (SD) age 54.3 (15.0) years, 79.8% female, were evaluated. Mean TBUT of 3.0 (2.6) sec and mean OSDI score of 36.5 (21.0) indicated severe DED at diagnosis. Most patients (87.3%) had a TBUT ≤5 sec, fulfilling ADES diagnostic criteria. Short TBUT (≤5 sec) and OSDI ≥13 had high sensitivity for diagnosing DED (87.3% and 90.1%, respectively), whereas an abnormal Schirmer’s test (69.5%) and abnormal CFS (42.3%) were less sensitive. MGD was diagnosed in 56.3% of patients. Dry eye-related characteristics in the non-short TBUT (>5 sec) group diverged for objective but not subjective clinical tests. Most common first treatments were artificial tears (95.8%) and corticosteroids (85.0%).

**Conclusions:**

DED diagnosis in routine hospital practice in Taiwan is highly concordant (87.3%) with ADES diagnostic criteria. TBUT appears to be an effective diagnostic tool for identifying dry eye in patients across symptom severity, etiology and age.

## Introduction

Dry eye disease (DED) is a common chronic inflammatory condition that may be initiated by numerous extrinsic or intrinsic factors which promote an unstable and hyperosmolar tear film. DED is characterized by symptoms of discomfort (e.g. persistent burning or irritation) and visual disturbances, with potential of damage to the ocular surface if left untreated [1–3]. Common risk factors for DED include advanced age, female sex, ambient environment (e.g., low temperature, low humidity, increased airflow over the surface of the eye), systemic medications, chronic autoimmune disorders (notably, Sjögren’s syndrome), and prolonged use of visual display terminals (VDT) [2−5].

The prevalence of DED ranges from 5 to 50% worldwide. In general, DED is more common in Asian than Caucasian populations, likely due to ethnic characteristics [6]. A meta-analysis estimates the pooled prevalence of DED in Asia to be approximately 20% [7]. In Taiwan, the prevalence of DED in the general population is approximately 5% [8] but increases to 33.7% in the subset over 65 years of age [9]. Epidemiological studies show that the crude incidence of DED in Taiwan increased steadily from 2001 to 2015 [10], underscoring the urgent need for effective diagnosis and treatment.

The first consensus guidelines of the Asia Dry Eye Society (ADES) published in 2017 emphasized tear film instability as the pivotal mechanism causing symptoms and/or visual impairment [11]. A follow-up report in 2020 focuses on the detection of DED and proposes a simple and practical approach based on the tear film breakup pattern using fluorescein [12]. The ADES consensus group regards the combination of patient-reported symptoms and fluorescein Tear Break Up Time (TBUT) ≤ 5 sec as sufficient to make a definitive diagnosis of dry eye [11, 12].

Despite these recommendations and simplified approach, there is no consensus at present about dry eye diagnosis or treatment in Taiwan. Due to health insurance regulations, an abnormal Schirmer’s test has been the criterion for artificial tear reimbursement over the past decade, prompting most ophthalmic institutions to routinely use this test for DED screening. Although the Schirmer test is a simple technique for diagnosing DED, its shortcomings include low sensitivity and specificity, poor reproducibility and patient discomfort if it is performed without topical anesthesia [13].

Fluorescein TBUT appears to be the next step in the evolution of dry eye diagnosis. However, before adopting the test as a diagnostic criterion of choice in local clinical practice, it is critical to understand the relationship between TBUT and the current diagnostic settings. This study aimed to evaluate real-world clinical treatment practice of DED in a hospital-based setting by analyzing whether existing diagnoses of DED fulfilled the ADES diagnostic criteria.

## Methods

This was a noninterventional, multicenter, cross-sectional, retrospective study of adults with newly diagnosed DED recruited from three tertiary hospitals in Taiwan: Taipei Veterans General Hospital, Far Eastern Memorial Hospital, and Kaohsiung Medical University Hospital. All medical procedures at participating sites were conducted in accordance with routine practice. Available clinical data collected from patients’ medical records included demographics, medical histories, dry eye-related characteristics and evaluation results, ocular conditions, dry eye symptoms, and dry eye treatment.

Included in the analysis were male and female adult outpatients aged ≥ 20 years with a new diagnosis of DED within 6 months of providing written informed consent. If both eyes were confirmed to have dry eye, the eye with the higher ocular surface staining score was selected. If both eyes had similar ocular surface staining scores, the right eye was selected. Excluded from analysis were patients with ocular conditions that could confound the diagnosis of DED; were unable to confirm subjective symptoms of dry eye; were illiterate and/or could not understand the dry eye questionnaire appropriately; had participated in other interventional trials in the previous 30 days; or were considered ineligible at the investigator’s discretion.

### Assessments

In hospital-based practice in Taiwan, a diagnostic work-up of dry eye typically involves assessment of the presence of symptoms (e.g. burning, discomfort, dryness sensation, grittiness, and blurred vision), a Schirmer’s test, and a TBUT test. A dry eye diagnosis is often made when TBUT is ≤ 5 sec, or the Schirmer’s test is abnormal. Ocular surface staining may be used as a supporting indicator but it is not a mandatory criterion. As the study reflected real-world practice, classification of dry eye subtype (aqueous deficient, evaporative, mixed) and dry eye severity (mild, moderate, severe), and diagnosis of meibomian gland dysfunction (MGD) were at the principal investigator’s discretion. Dry eye subtype classification was based mainly on Schirmer’s test results and meibomian gland evaluation.

TBUT was measured as described in the ADES consensus report [11]. After instilling a small quantity (< 2 µL) of fluorescein dye, the patient was instructed to blink three times to ensure adequate mixing with tears. The time interval between the last blink and appearance of the first dark spot on the cornea was measured by stopwatch. The mean value of three measurements was used. A cutoff value of ≤ 5 sec signals a diagnosis of dry eye.

Patients’ symptoms were assessed using Chinese language versions of the ocular surface disease index (OSDI), a 12-item patient-reported questionnaire scored from 0 to 100 (https://osdi.com.tw/), with higher scores representing greater ocular disability; and the Standard Patient Evaluation of Eye Dryness (SPEED), an 8-item questionnaire scored from 0−28, with higher scores representing greater symptom severity.

Dry eye severity based on CFS was evaluated according to the National Eye Institute (NEI) or Oxford grading systems. The NEI grading scale (0–15) defines a CFS score of 0–2 as absent, 3–6 as mild, 7–9 as moderate, and 10–15 as severe [14]. The Oxford grading scale (0–5) defines a CFS score of 0 as absent, 1–2 as mild, 3 as moderate, and 4–5 as severe [15]. For analysis purposes, the scales were unified and simplified to report severity as absent, mild, moderate, and severe.

Schirmer’s test, which measures tear production, differentiates between methods with and without anesthesia. The basic Schirmer’s test with anesthesia considers ≤ 5 mm of wetting after 5 min as abnormal (i.e. insufficient tear production). The Schirmer I test without anesthesia defines < 10 mm of wetting in the same 5-minute duration as abnormal [16].

MGD was diagnosed according to meibomian gland expressibility and meibum quality [17, 18]. MGD was identified when some glands were inexpressible or the meibum was cloudy or inspissated; at some study sites, both conditions had to be met for diagnosis. When available, the tear film break-up pattern, lipid layer thickness (< 60 nm), and meibography were utilized to refine the diagnosis, particularly in cases with borderline expressibility or meibum quality abnormalities. Due to the extra time required and associated costs, these tests were not routinely performed and the diagnostic criteria were adjusted accordingly.

### Outcomes

The primary outcome measure was the proportion of patients diagnosed with DED in current hospital-based practice who fulfilled ADES criteria for DED. A patient was determined to have met the ADES diagnostic criteria if he/she had satisfied both criteria: presence of dry eye symptoms and fluorescein TBUT ≤ 5 sec. Other outcomes included DED classification, DED severity, CFS severity, Schirmer’s test (with or without anesthesia), OSDI score, SPEED score, and presence of MGD. Data regarding the first treatment modality for DED were also collected.

### Ethical approval

The study was approved by the Institutional Review Board at each study site and was performed in accordance with the study protocol, Good Clinical Practice, any applicable privacy regulations, International Conference on Harmonization guidelines, applicable regulations and guidelines governing clinical study conduct, and ethical principles that have their origin in the Declaration of Helsinki. Written informed consent was obtained from each patient.

### Statistical analysis

Data from all eligible patients were analyzed descriptively. Continuous variables are summarized by means and standard deviations (SDs). Categorical variables are summarized by counts and percentages. McNemar tests were used for pairwise comparisons of the sensitivity of clinical tests in patients with DED. To compare outcomes between short (≤5 sec) and non-short (>5 sec) TBUT groups, Chi-square tests were used for categorical data, and independent t tests were used for continuous data. Associations between DED-related test results and age groups or DED type were analyzed using a chi-square test. A P value <0.05 was considered statistically significant by two-sided tests. All statistical analyses were performed using IBM SPSS (Version 26.0. IBM Corp.).

## Results

The data collection period was from 22 October 2021 to 28 February 2022. Data from 213 patients with newly-diagnosed DED were included in the analysis. Clinical characteristics of the population as a whole and of subgroups stratified according to short or non-short TBUT are summarized in Table 1. Mean (SD) age was 54.3 (15.0) years and 79.8% of the population was women. The most common DED subtypes were mixed type (38.5%) and aqueous tear deficiency type (37.1%). Two-thirds (66.2%) of patients had ocular comorbidities, including cataracts (97 cases), conjunctivitis (21 cases), glaucoma (14 cases), and retinal diseases (13 cases). More than half of patients (56.3%) were diagnosed with MGD.

**Table 1:** Clinical characteristics of DED patients stratified by short TBUT and non-short TBUT status

Parameter	All patients (*N* = 213)	Short TBUT (*n* = 186)	Non-short TBUT (*n* = 27)	*P* value*
Age (years), mean ± SD	54.3 ± 15.0	54.1 ± 14.7	55.4 ± 17.0	0.690
Female sex, n (%)	170 (79.8)	150 (80.6)	20 (74.07)	0.427
Use of contact lens, n (%)	32 (15.0)	25 (13.4)	7 (25.9)	0.144
Work with video display terminal, n (%)	97 (45.5)	82 (44.1)	15 (55.6)	0.263
Type of dry eye subtype, n (%)				
Aqueous deficient	79 (37.1%)	65 (34.9)	14 (51.9)	0.007
Evaporative	39 (18.3%)	30 (16.1)	9 (33.3)	
Mixed	82 (38.5%)	79 (42.5)	3 (11.1)	
Unclassified	13 (6.1%)	12 (6.5)	1 (3.7)	
Severity of dry eye, n (%)				
Mild	134 (62.9)	112 (60.2)	22 (81.5)	0.101
Moderate	62 (29.1)	58 (31.2)	4 (14.8)	
Severe	17 (8.0)	16 (8.6)	1 (3.7)	
Ocular comorbidities, n (%)	141 (66.2)	119 (64.0)	22 (81.5)	0.073
Ongoing systemic comorbidities, n (%)	74 (34.7)	60 (32.3)	14 (51.9)	0.046
Previous ocular procedure, n (%)	32 (15.0)	26 (14.0)	6 (22.2)	0.386
CFS (severity)‡, n (%)				
Absent	123 (57.7)	107 (57.5)	16 (59.3)	0.632
Mild	79 (37.1)	68 (36.6)	11 (40.7)	
Moderate	7 (3.3)	7 (3.8)	0 (0.0)	
Severe	4 (1.9)	4 (2.2)	0 (0.0)	
TBUT (sec), mean ± SD	3.0 ± 2.6	2.2 ± 1.3	8.9 ± 1.7	< 0.001
Schirmer’s test result, n (%)				
Abnormal	148 (69.5)	134 (72.0)	14 (51.9)	0.033
Normal	65 (30.5)	52 (28.0)	13 (48.1)	
OSDI value (0–100), mean ± SD	36.5 ± 21.0	36.5 ± 21.0	35.9 ± 21.2	0.885
SPEED score (0–28), mean ± SD	12.0 ± 6.0	11.8 ± 6.0	13.6 ± 5.8	0.144
Meibomian gland dysfunction (MGD), n (%)				
MGD diagnosis	120 (56.3)	109 (58.6)	11 (40.7)	0.081

*CFS* corneal fluorescein staining, *OSDI* ocular surface disease index, *SPEED* standardized patient evaluation of eye dryness, *TBUT* tear breakup time

*Short TBUT (≤ 5 sec) vs. non-short TBUT (> 5 sec) groups

‡ NEI grading scale (0–15): CFS = 0–2, absent; CFS = 3–6, mild; CFS = 7–9, moderate; CFS = 10–15, severe; or Oxford grading scale (0–5): CFS = 0, absent; CFS = 1–2, mild; CFS = 3, moderate; CFS = 4–5, severe. For analysis purposes, the scales were unified and simplified to report severity as absent, mild, moderate, and severe

In all, 186 (87.3%) of 213 patients had a TBUT ≤5 sec which satisfied the ADES diagnostic criteria for DED (Table 1).

Statistically significant differences were observed between short and non-short TBUT groups in some clinical characteristics (Table 1). Short TBUT patients were more likely than non-short TBUT patients to have a mixed type diagnosis (42.5% vs 11.1%, P = 0.007). Fewer short TBUT than non-short TBUT patients had systemic comorbidities such as diabetes, rheumatoid arthritis, and Sjögren’s syndrome (32.3% vs 51.9%; P = 0.046). A greater proportion of short than non-short TBUT patients had an abnormal Schirmer’s test result (72.0% vs 51.9%; P = 0.033). All patients were symptomatic as per OSDI and SPEED scores, with significant differences observed between the short and non-short TBUT groups.

TBUT ≤5 sec and OSDI ≥13 were both highly sensitive for diagnosing DED (87.3% and 90.1%, respectively) with no significant difference between these clinical tests (P = 0.461). An abnormal Schirmer’s test and a CFS >0 were less sensitive for diagnosing DED, with all pairwise comparisons (McNemar tests) to TBUT ≤5 sec and OSDI ≥13 reaching statistical significance (P < 0.001) (Table 2).

**Table 2:** Pairwise comparisons (McNemar tests) of clinical test sensitivity in patients with dry eye disease (*N* = 213)

	*n*	% (95% CI)	*P* value: A vs.	*P* value: B vs.	*P* value: C vs.
A) Short TBUT (≤ 5 sec)	186	87.3 (82.8 to 91.8)	–	–	–
B) Abnormal Schirmer’s test	148	69.5 (63.3 to 75.7)	< 0.001	–	–
C) CFS > 0	90	42.3 (35.6 to 48.9)	< 0.001	< 0.001	–
D) OSDI ≥ 13	192	90.1 (86.1 to 94.1)	0.461	< 0.001	< 0.001

*CFS* corneal fluorescein staining, *OSDI* ocular surface disease index, *TBUT* tear breakup time

In patients who satisfied the ADES diagnostic criteria for DED (n = 186), the likelihood of an abnormal Schirmer’s test increased with advancing age (P = 0.028, by trend test). Age had no significant association with TBUT, CFS or ODSI test results (Table 3).

**Table 3:** Status of clinical tests for dry eye disease according to age group

Parameter	Age ≤ 40 years (*n* = 42)	Age 40 to ≤ 60 years (*n* = 83)	Age > 60 years (*n* = 88)	*P* value
TBUT, n (%)				
≤ 5 sec	37 (88.1)	74 (89.2)	75 (85.2)	0.732
> 5 sec	5 (11.9)	9 (10.8)	13 (14.8)	
Schirmer’s test, n (%)				
Abnormal	24 (57.1)	57 (68.7)	67 (76.1)	< 0.028*
Normal	18 (42.9)	26 (31.3)	21 (23.9)	
CFS, n (%)				
Absent	30 (71.4)	48 (57.8)	45 (51.1)	0.300
Mild	11 (26.2)	29 (34.9)	39 (44.3)	
Moderate	1 (2.4)	3 (3.6)	3 (3.4)	
Severe	0 (0.0)	3 (3.6)	1 (1.1)	
OSDI, n (%)				
Normal (0–12)	5 (11.9)	5 (6.0)	11 (12.5)	0.192
Mild (13–22)	5 (11.9)	12 (14.5)	21 (23.9)	
Moderate (23–32)	9 (21.4)	14 (16.9)	18 (20.5)	
Severe (33–100)	23 (54.8)	52 (62.7)	38 (43.2)	

*CFS *corneal fluorescein staining, *OSDI *ocular surface disease index, *TBUT* tear breakup time

*Cochran-Armitage test for trend

In patients who satisfied ADES diagnostic criteria for DED (n = 186), concordance was high (>76.9%) between a short TBUT and all DED types (P = 0.007), although was highest for the mixed type dry eye (96.3%). Concordance between an abnormal Schirmer’s test and DED type was significant (P < 0.001) for mixed type (96.3%) or aqueous tear deficiency type (87.3%), but not evaporative type. There was no significant association between DED type and CFS or ODSI test results (Table 4).

**Table 4:** Status of clinical tests for dry eye disease (DED) according to DED type

Parameter	Aqueous tear deficiency (*n* = 79)	Evaporative (*n* = 39)	Mixed (*n* = 82)	Unclassified (*n* = 13)	*P* value*
TBUT, n (%)					
≤ 5 sec	65 (82.3)	30 (76.9)	79 (96.3)	12 (92.3)	0.007
> 5 sec	14 (17.7)	9 (23.1)	3 (3.7)	1 (7.7)	
Schirmer’s test, n (%)					
Abnormal	69 (87.3)	0 (0.0)	79 (96.3)	0 (0.0)	< 0.001
Normal	10 (12.7)	39 (100.0)	3 (3.7)	13 (100.0)	
CFS, n (%)					
Absent	46 (58.2)	20 (51.3)	45 (54.9)	12 (92.3)	0.298
Mild	27 (34.2)	18 (46.2)	33 (40.2)	1 (7.7)	
Moderate	3 (3.8)	1 (2.6)	3 (3.7)	0 (0.0)	
Severe	3 (3.8)	0 (0.0)	1 (1.2)	0 (0.0)	
OSDI, n (%)					
Normal (0–12)	6 (7.6)	5 (12.8)	9 (11.0)	1 (7.7)	0.902
Mild (13–22)	14 (17.7)	5 (12.8)	16 (19.5)	3 (23.1)	
Moderate (23–32)	12 (15.2)	8 (20.5)	18 (22.0)	3 (23.1)	
Severe (33–100)	47 (59.5)	21 (53.8)	39 (47.6)	6 (46.2)	

*CFS* corneal fluorescein staining, *OSDI* ocular surface disease index, *TBUT *tear breakup time

*Chi-square test

The most common first treatment modality for DED patients was artificial tears (95.8%), followed by corticosteroids (85.0%) (Table 5).

**Table 5:** First treatment modality in patients with dry eye disease (*N* = 213)

Treatment	*n* (%)
Artificial tears	204 (95.8)
Corticosteroids	181 (85.0)
Other	11 (5.2)
Mucin secretagogues	10 (4.7)
Serum	3 (1.4)
Antihistamines	2 (0.9)
Antibiotics	2 (0.9)

## Discussion

The primary aim of this study was to investigate whether new diagnoses of DED in hospital-based settings in Taiwan fulfilled the ADES diagnostic criteria and to describe the demographics and clinical characteristics of newly-diagnosed patients. We found 87% concordance between hospital-based practice for DED diagnosis and the ADES diagnostic criterion of TBUT ≤5 sec. Aligning with similar multicenter observational studies conducted in Japan (the DECS-J study [19]) and Korea (the DECS-K study [20]), a short TBUT appears to be a signal of DED irrespective of country or care setting (clinic or hospital). In DECS-J, 94.9% of 449 patients with DED who visited eye clinics had a TBUT ≤5 sec, regardless of dry eye subtype [19]. In DECS-K, 94.3% of 158 patients who were diagnosed with DED in hospital settings had a TBUT ≤5 sec [20]. In the Osaka study in relatively young VDT users (n = 561), 78.6% of participants had a TBUT ≤5 sec, even in the absence of abnormal tear secretion or obvious ocular surface staining [5]. Collectively, these studies support the use of tear film instability as a diagnostic criterion for DED in clinical practice.

Our patient sample was representative of DED populations in similar studies in terms of age and female preponderance [19, 20]. Patients presented severe DED, based on a mean TBUT of 3.0 sec and a mean OSDI of 36.5 at diagnosis. Consistent with a large single-center study of 4817 Taiwanese patients with DED [21], in our cohort, aqueous deficient type dry eye was two-fold more common than evaporative type dry eye (37.1% vs 18.3%). This may reflect the tertiary hospital setting which tends to see more patients with Sjögren’s syndrome and autoimmune diseases associated with aqueous deficient type dry eye.

Whereas dry eye-related clinical characteristics between the short TBUT group and the population as a whole were similar, the non-short TBUT group showed distinct outcomes in objective dry eye assessments (e.g. TBUT, Schirmer’s test) but similar outcomes in subjective assessments (e.g. OSDI, SPEED), highlighting the inconsistency of subjective evaluations in diagnosing DED.

Among objective indicators of dry eye, TBUT had a significantly higher sensitivity than an abnormal Schirmer’s test (87.3% vs 69.5%) for diagnosing DED. TBUT is influenced by all tear components (lipid, mucin and aqueous), whereas the Schirmer’s test is affected mainly by the aqueous component. Furthermore, Schirmer’s test is a poor discriminator of milder forms of DED [22]. Due to the Taiwanese health insurance reimbursement scheme regulations, almost all patients evaluated for dry eye undergo a Schirmer’s test. In our study, Schirmer’s test protocols varied between hospitals and included both anesthetized and nonanesthetized tests. The data suggest that the presence of normal component-specific parameters indicated by normal Schirmer’s test results does not necessarily indicate good tear film stability. Conversely, TBUT ≤5 sec is an effective and primary diagnostic indicator of DED because it reflects tear film instability, a major factor in the pathogenesis of DED [11]. The sensitivity of OSDI ≥13 as an indicator of DED warrants closer examination given the lack of difference in mean OSDI scores between the short and non-short TBUT groups.

The clinical diagnosis of DED focusses on dry eye classification, indicating widespread acceptance of the concept of tear film-oriented therapy [23]. However, due to limitations in testing techniques in clinical practice, the main distinction continues to be between the aqueous and evaporative dry eye types. A recent retrospective study revealed that dry eye without a definitive pathophysiology accounted for approximately 20% of cases in which unstable tear film could be associated with mucin deficiency [21]. Our study identified a small subgroup (6.1%) of patients with unstable tear film but no abnormalities in the aqueous or lipid components, resulting in their categorization as unclassified. Additionally, we observed that results from the Schirmer test and MGD assessments did not always align with the categorization of dry eye types. These discrepancies may be attributed to factors such as test reliability, variability in investigators’ perceptions of severity, and inconsistencies in test results. Moreover, physicians’ judgments regarding the primary cause of dry eye may vary based on the patient’s overall condition.

Perception differences may also occur in investigators’ discrimination of MGD. Although 56.3% of patients were diagnosed with MGD, the proportion of MGD diagnoses varied significantly among study sites (data not shown). This discrepancy is further complicated by the diverse criteria used to diagnose MGD. To illustrate, the condition could be overestimated when even slightly poor meibum quality is used to determine MGD or it could be underestimated when a cutoff value of ≤60 nm lipid layer thickness (LLT) measured by LipiView® is applied, especially in middle-aged and older individuals. In these cases, sebum in the dermatochalasis skin folds may contribute to an increased LLT [24]. Unified diagnostic standards for MGD across multiple research locations are required to ensure consistency and reliability in the classification of dry eye conditions.

In line with ADES diagnostic criteria, McMonnies argues that a short TBUT might occur independently of lipid and/or mucin deficiency, and even in the presence of aqueous deficiency [25]. Clinically, when problems with aqueous or mucin components go undetected, there may be an overemphasis on diagnosing MGD. In our study, non-short TBUT patients comprised 13% of the population and had been diagnosed clinically with DED despite not meeting ADES criteria. Relative to the short TBUT group, patients in the non-short TBUT group had milder DED, consistent with their TBUT test results, and a lower incidence of mixed type dry eye indicating a tendency towards the pre-clinical stage of DED. Approximately half of the non-short TBUT group had abnormal Schirmer’s test results and, despite relatively mild or absent signs of DED, their symptoms as reflected by mean OSDI values were comparable to those of the short TBUT group. These observations suggest that in real-world practice, when tear film instability is borderline, abnormalities detected by the Schirmer test, staining, and MGD are emphasized in the diagnosis of dry eye.

An examination of the relationship between clinical characteristics and diagnostic tests revealed a significant increase in the proportion of abnormal Schirmer’s tests with advancing age (P < 0.01). Trends toward increased severity of dry eye with advancing age were evident in the distribution proportions for CFS and OSDI but did not reach statistical significance. A short TBUT and an abnormal Schirmer’s test were most likely to occur in patients with aqueous deficient or mixed type dry eye.

In this dry eye study, main first-line treatments in tertiary hospitals were artificial tears (95.8%) and corticosteroids (85.0%). Of note, although a proportion of patients with DED will eventually receive cyclosporine (CsA) after failure of corticosteroid treatment, Taiwan’s reimbursement policy does not allow CsA as a first-line treatment [26]. In Japan, hyaluronic acid (66%) and diquafosol (40%) eye drops are the most common treatments used for DED [19]. In Korea, the most common treatments for DED are also hyaluronic acid (34%) and diquafosol (15%), with the latter used mainly to treat evaporative dry eye and mild cases of DED [20]. Interestingly, the Osaka cross-sectional study revealed low mean tear mucin 5AC (MUC5AC) concentrations in the early stages of dry eye in young VDT workers [27]. This highlights the potential for developing mucin layer treatments for early-stage disease.

The study has certain limitations. The COVID-19 pandemic reduced the anticipated number of participants which impacted data analysis due to small sample sizes in some data sets. Although the tertiary hospital setting allowed for collection of TBUT outcomes, it may have introduced a source of bias as patients with preclinical or early-stage disease are more likely to visit clinics. Furthermore, because MGD diagnostic criteria are not standardized across tertiary hospitals, the results are variable and represent a study limitation. While the general approach to diagnosing DED was similar across study sites, the nature of real-world studies is that investigators are at liberty to use their discretion. For example, there may have been flexibility in applying cut-off values for clinical tests or, in the event of inconclusive clinical tests, a patient’s symptoms (especially if severe) may have been considered sufficient to arrive at a diagnosis. Patients were identified for the study based on a DED diagnosis in their medical charts, and all relevant clinical parameters were collected accordingly. The DED diagnosis was not verified in each patient as this is not required for a real-world study and is unrealistic in the context of real-world practice in Taiwan. Due to local reimbursement policy, TBUT is not routinely conducted for dry eye diagnosis in clinic settings in Taiwan. Introducing TBUT into clinics for patients with mild disease would enable targeted treatment to be delivered.

This study represents Taiwan’s first multicenter dry eye survey, eliminating potential bias from single-center studies and providing crucial baseline information on the demographics, dry eye characteristics and first treatment modalities of patients with newly diagnosed DED. Our results suggest that DED diagnosis in routine hospital practice has a high degree of concordance (87.3%) with ADES diagnostic criteria, demonstrating the ease of use of a fluorescein-based diagnosis and the relevance of TBUT as a method for diagnosing DED. Patients who failed to satisfy the ADES diagnostic criteria for dry eye (i.e. had a TBUT >5 sec) showed distinct outcomes in objective dry eye assessments but similar outcomes in subjective dry eye assessments, illustrating disparities between objective and subjective dry eye evaluations in real-world settings. However, due to the limited number of patients, the results should be interpreted with caution. Future studies involving more DED patients, including those undergoing cataract surgery, are needed to gain wider insight into the real-world dry eye profile in Taiwan.
